# The Micrococcus of Bluish Pus

**Published:** 1880-08

**Authors:** P. W. Van Peyma


### THE MICROCOCCUS OF BLUISH PUS.

FROM THE DUTCH BY P. W. VAN PEYMA, M. D.

As is well known, a controversy has long existed between Cohn, on the one hand, and Billroth and Naegele, on the other, over the question whether, in the case of the Schizomycetes, there exist as many distinct species as variations in form; or whether the various forms are capable of transition into each other, thus proving the existence of but one, or at least of but very few species. This controversy is being vigorously pushed by Cohn, and he has mentioned the micrococcus prodigiosus and luteus as examples whose form and color are permanently maintained, no matter how the coccus is propagated. He adds that if by propagation other varieties develop they must be considered as arising, not from the micrococcus prodigiosus or luteus, but rather as having a foreign source; should, however, cocci of similar forms and color appear, this is to be considered as conclusive proof of the stability of the various forms of Schizomycetes. Strange as it may seem this argument has been allowed to have force with even some quite intelligent persons.

Some experiments tending to answer this question have been made by Dr. Kurlich's in Langenbeck's Archives in an article entitled, "Ueber Vegetation von Rigment Bacterian in Verbandstoffen."

The investigations seem to have been made under the supervision of Prof. Von Bergman, of Wurzburg, who is well known as having written upon this and similar subjects. The investigations were limited to the varieties of coccus found in bluish pus. The cocci are recognizable by a peculiar aromatic sweetish odor. Von Bergman recognized this odor in the sputa of a patient with pulmonary gangrene. The colorless coccus inoculated upon purulent granulations caused bluish pus. As the result of a gunshot wound, a patient had a pus-secreting wound of the tongue and lower maxillary; the secretion, not bluish, had the peculiar odor, and by inoculation upon a purulent granulating surface, the blue pus cocci were formed.

The color and nature of the cocci have, therefore, but an indirect connection with each other. This is further shown by the fact that these cocci can be made to increase in Pasteur's fluid. They are here entirely colorless but can be made to assume the blue color upon mixing with pus. The yellow cocci which in Wurzburg is often found upon moistened bread exposed to the air, when added to pus, were transformed into the blue variety. The blue pus cocci, the yellow cocci of bread, the colorless offspring of the blue cocci in the fluid of Pasteur when implanted upon gypsum produce red cocci. Still, in other ways was it determined that the color is really of no moment to the cocci. Cohn would thus appear to have no reason for distinguishing the colored cocci from the colorless. In no instance does it seem warranted to classify according to color.—*Weeksblad von het Nedelandsch. Tydschrift von Geneeskunde.*

